# Water and Sanitation Hygiene Knowledge Attitude Practice in Urban Slum Settings

**DOI:** 10.5539/gjhs.v6n2p23

**Published:** 2013-11-18

**Authors:** Ashish Joshi, Satish Prasad, Jyoti B Kasav, Mehak Segan, Awnish K Singh

**Affiliations:** 1 Center for Global Health and Development, College of Public Health, University of Nebraska Medical Center, Omaha, USA; 2 Foundation of Healthcare Technologies Society, New Delhi, India

**Keywords:** water, sanitation, hygiene, practices, urban slum

## Abstract

**Background:**

Access to improved drinking water, sanitation and hygiene is one of the prime concerns around the globe. This study aimed at assessing water and sanitation hygiene-related attitude and practices, and quality of water in urban slums of south Delhi, India.

**Methodology:**

This pilot cross sectional study was performed during July 2013 across four urban slums of South Delhi. A convenient sample of 40 participants was enrolled. A modified version of previously validated questionnaire was used to gather information on socio-demographics, existing water and sanitation facilities and water treatment practices. Water quality testing was additionally performed using hydrogen sulphide (H_2_S) vials.

**Results:**

Average age of participants was 36 years (SD=10). 83% of the participants perceived gastrointestinal tract infection as the most important health problem. 75% of the participants did not use any method for drinking water treatment. 45% of the participants consumed water from privately-owned tube well/bore well. Water shortage lasted two days or more (50%) at a stretch with severe scarcity occurring twice a year (40%). Females aged 15 years and above were largely responsible (93%) for fetching water from water source. 45% of the participants had toilets within their households. 53% of drinking water samples collected from storage containers showed positive bacteriological contamination.

**Discussion:**

There is an urgent need to develop family centered educational programs that would enhance awareness about water treatment methods that are cost effective and easily accessible.

## 1. Background

Improving drinking water condition and sanitation facilities remains a major concern globally. Though 89% of the world’s population has access to drinking water facilities, about 768 million people rely on unimproved drinking water-sources; 83% of them residing in rural areas (World Health Organization/United Nations International Children’s Emergency Fund [WHO/UNICEF], 2013; United Nations Millennium Development Goals [UN MDGs], 2013).

1.9 billion people gained access to improved sanitation facility over a period of two decades (1990 to 2011) with an average rate of 240,000 individuals gaining access every day. 8% of Indian population is still devoid of clean water, and only 25% of population have access to piped water in premises (WHO/UNICEF Joint Monitoring Programme [JMP], 2013). This current growth rate is not enough to achieve sanitation target (Target 7.C: Halve, by 2015, the proportion of the population without sustainable access to safe drinking water and basic sanitation) of Millennium Development Goals (MDGs). It is predicted that a daily average increase of 660,000 individuals per day have to be provided with improved sanitation facilities till 2015 if the set target has to be achieved (UN MDGs, 2013). However, the current trend is showing a gap of 11% from the expected target of MDGs to be achieved by 2015 (UN MDGs, 2013).

According to previous study, burden of diseases on world could be decreased by 10% through prevention by improvement in the quality of drinking water and water resource management simultaneously with sanitation and hygiene (WHO/UNICEF JMP, 2013). A study by the World Health Organization (WHO) shows that to meet Target 7C of the MDGs, an investment of US$11.3 billion per year can give a payback of US$84 billion (Pruss-Ustun et al., 2008; Hutton & Haller, 2004).

Sanitation is also one of the major environmental health issues to be addressed. The Joint Monitoring Project (JMP) report of 2013 estimates that half of the Indian population still practice open defecation. A meager 17% increase in sanitation facilities to the target population since 1990 (UN MDGs, 2013), can be related to the growth in the population or density of slum dwellers, especially in South Eastern Asia (UNICEF/MDGs progress chart, 2013). Out of 2.5 billion diarrheal cases occurring every year among under-five children, more than half occur from Africa and South Asia. The total death toll due to diarrhea is about 1.5 million every year globally. The disease is more severe as it causes more deaths than AIDS, malaria and measles combined in young children (WHO statistics, 2013). Diarrhea is also one of the leading causes of under nutrition in children with diarrheal disorders causing 12.6% of total deaths in children under the age group five years (Chambers & von Medezza, 2013).

Chlorination and candle filtration are cost effective methods used commonly in household to reduce diarrheal illness by 30%. Better advanced technologies can reduce this proportion by 70% (Clasen et al., 2007; Fewtrell et al., 2005). Poor water and sanitation conditions in India not only take a toll on health of population and mortality, but also cause a major loss to economy. The impact of improper sanitation alone accounts for 2.44 trillion rupees (US$ 53.8 billion) every year, which was equal to 6.4% of India’s Gross Domestic Product (GDP) in 2006 (Water and Sanitation Programme [WSP], 2011).

Inadequate knowledge and poor practices of storing drinking water can cause severe effects on health of the population. For reduction of water borne diseases, there is a need to understand the current trend of attitudes and practices of individuals living in urban slums.

The objective of this pilot study was thus to explore water and sanitation hygiene-related attitudes and practices among individuals living in urban slum settings of south Delhi, India.

## 2. Methodology

This was a pilot cross sectional study conducted in New Delhi, India during July 2013. Four urban poor slums were selected conveniently based on proximity and cooperation from the community. From the four slums, a convenient sample of 40 participants was taken. Individuals aged 18 years and above, residing in the urban poor slum and giving informed consent were included in the study. Those individuals with mental or physical challenges making it difficult for them to participate in the study or involved in clinical trials during the study period were excluded from the study.

The study protocol was approved by the Institutional Review Board (IRB) of Foundation of Healthcare Technologies Society (FHTS) with the IRB reference number of IRB#FHTS/034/2013. Written informed consent was obtained from the individuals after the study’s purpose was explained to them. Confidentiality of the participants was maintained by assigning a unique code to each of the participants.

For data collection, households from each of the slums were selected by simple random technique. A sampling frame of all households containing at least one participant aged 18 years or above in each slum was prepared. Efforts were made to interview the head of the household. If the head of the household was unavailable, the spouse was interviewed. In the event of absence of both the husband and the wife, the next immediate resident aged 18 years and above was interviewed. If the household was locked or no eligible participant was found at the time of interview, the household was either revisited on the subsequent day or an additional household was chosen. All interviews were conducted during the day time.

Data was collected by a modified version of previously validated questionnaire. The WHO and United Nations Children’s Fund (UNICEF) ‘Core questions on drinking-water and sanitation for household surveys’ was contextually modified and used. This questionnaire consists of a set of harmonized questions widely used by nations in their surveys to make data accurate and comparable across the globe (WHO/UNICEF, 2006). Information was gathered on the following variables:

(a) **Socio-demographic characteristics**: Information was gathered about age (years), gender, educational status [primary school (1-5 grade), secondary (6-8 grade), high school (9-10 grade), intermediate (11-12 grade) or post high school diploma, graduate or post graduate, professional or honors, illiterate)], marital status (single/married/divorce or separated/widow), family income categories (≤1520/1521-4555/4556-7593/7594-11361/11362-15187/15188-30374/≥30375 INR) (Sharma et al., 2012), type of family (joint, nuclear, broken, extended), number of family members, and occupation status (professional, semi-professional, clerical, shop owner, farmer, skilled worker, semi skilled worker, unskilled worker, unemployed).
(b) **Water facility and use:** Information was gathered about the various sources of drinking water, individuals that were responsible for fetching of water in the household, water shortage periods, distance of water source from household, timings of water supply and water storage practices.
(c) **Water Treatment:** Information was gathered about individuals’ attitudes towards water treatment practices. These variables included topics related to water safety, effects of unsafe drinking water on health, and the practices that were adopted to make water safe to drink.
(d) **Sanitation:** Information was also gathered about toilet facilities, hand washing, and waste disposal facilities.

### 2.1 Water Quality Testing

Water samples were collected for testing the bacteriological contamination. This was done by using Hydrogen Sulphide (H_2_S) vials to test the presence of pathogenic bacteria which causes common water borne diseases like diarrhoea, dysentery and gastroenteritis. TARAenviro Aquacheck H_2_S vials (New Delhi), based on UNICEF guidelines were used (TARAenviro, http://www.taraenviro.com/?page_id=72).

Procedure: Drinking water samples were directly collected into H_2_S vials from drinking water storage containers. Water was collected up to the 20 ml mark on the vials, shaken well and incubated for 48 hours at 25°C and protected from direct sunlight. Water pH and bacteriological contamination were assessed. After the incubation period, samples were checked for the presence or absence of contamination. Contamination was indicated if the water samples turned to blue black in colour after incubation period. No change in colour of the samples indicated an absence of any bacteriological contamination.

### 2.2 Statistical Analysis

Descriptive analysis was performed using univariate statistics to report means and standard deviations for the continuous variables and frequency distribution for the categorical variables. T statistics was performed to compare differences in the continuous variables and chi-square analysis was performed to compare the frequency of categorical variables. All analysis was performed using SPSS version 16.

## 3. Results

A total of 40 participants were enrolled in the study conducted in July 2013, in urban poor slum settings in Delhi, India.

Average age of the participants was 36 years (SD=9.7). Majority of the participants were females (73%, n=29), were married (95%, n=38), lived in nuclear families (68%, n=27) with an average family size of 6 (SD=2) and were educated up to high school level or less (83%, n=33). More than half of the study participants were unemployed (68%, n=27). The average family income was in the range of 4556-7593 INR (Table 1).

**Table 1:** Study participant characteristics

Socio-demographics	
Variables	Results
**Age (Years)**	Mean=36, SD=9.7
**Gender**	
Female	73% (n=29)
**Marital status**	
Married	95% (n=38)
**Family structure**	
Nuclear	68% (n=27)
Extended	20% (n=8)
Joint	12% (n=5)
**Family size**	Mean=6, SD=2.3
**Highest education level**	
No education	50% (n=20)
>High school	33% (n=13)
≤ High school	17% (n=7)
**Occupation**	
Unemployed	68% (n=27)
Skilled worker	15% (n=6)
Unskilled worker	7% (n=3)
Others	10% (n=4)
**Monthly family income (INR)**	
<1521	5% (n=2)
1521-4555	25% (n=10)
4556-7593	53% (n=21)
7594-11361	10% (n=4)
11362-15187	2% (n=1)
15188-30374	5% (n=2)

Majority of the participants (78%, n=31) perceived that the available water is safe for drinking. 95% (n=38) of the participants perceived that the quality of water can affect health. Majority of the participants (83%, n=33) perceived gastro intestinal tract infection to be the most important effect of consuming unsafe drinking water. 75% (n=30) of participants did not use any method to make water safe for drinking, the major reason being the perception that water received was already clean (73%, n=22).

All participants perceived that hands should be washed prior to handling of food. Other perceived critical times of hand washing were after defecation (88%, n=35) and after eating (75%, n=30) among other reasons. Almost all participants washed their hands before eating food (98%, n=39). Other times of hand washing included before handling food (90%, n=36), after defecation (88%, n=35) and after eating (75%, n=35). 78% (n=31) of the participants washed their hands because they perceived it was hygienic, and because it could prevent infection (75%, n=30). Almost all participants (98%, n=39) disposed their solid wastes in the community dustbin (Table 2).

**Table 2:** Water and sanitation hygiene attitudes and practices

Water and Sanitation Hygiene Attitudes and Practices	
Variables	Results
**Do you think the quality of water you receive is safe**	
Safe	78% (n=31)
Unsafe	22% (n=9)
**Does quality of water affect health**	
Yes	95% (n=38)
No	5% (n=2)
**Most common effects of using unsafe drinking water**	
Gastro intestinal tract disturbances	83% (n=33)
Fever	20% (n=8)
Other health problems	15% (n=6)
**Current methods being used to make water safe**	
Nothing	75% (n=30)
Filter	15% (n=6)
Boiling	10% (n=4)
**Reason for not treating to make water clean (N=30)**	
Water is already clean	73% (n=22)
Expensive methods	20% (n=6)
Don’t know methods of cleaning	7% (n=2)
**Challenges in procuring drinking water**	
No challenges	73% (n=29)
Distant source	18% (n=7)
Irregular supply	7% (n=3)
Conflict	2% (n=1)
**Sanitation Attitudes and Practices**	
**Perception about critical times of hand washing**	
Before handling food	100% (n=40)
After defecation	88% (n=35)
After food	75% (n=30)
After weaning/changing the baby	15% (n=6)
When entering home from outdoors	5% (n=2)
**Hand washing practices**	
Before eating	98% (n=39)
Before handling food	90% (n=36)
After defecation	88% (n=35)
After eating	75% (n=30)
**Reasons for hand washing**	
Hygiene: feel clean	78% (n=31)
Health: prevent infection	75% (n=30)
Appearance: appears good	2% (n=1)
Because everyone does	2% (n=1)
**Solid waste disposal**	
Community dustbin	98% (n=39)
In open drain	10% (n=4)
Burn in open	2% (n=1)

*some of the variables are in multiple responses

Results showed that piped water in yard/plot (45%, n=18) and bore well/tube well (30%, n=12) were among the main sources of water. 18% (n=7) availed water from both the sources. More than half of the water sources was through public supply (53%, n=21). 48% (n=19) had access to water source within the household. Results showed a gender disparity in fetching water, as females (93%, n=37) were largely responsible for fetching water. Majority of the participants had to walk a distance of ≤30 minutes to fetch water. 88% (n=35) of the study participants agreed that their water needs were met. 75% (n=30) of the study participants reported water supply to be available especially in the morning and evening times. 40% (n=16) of them indicated water shortage for about twice a year and half of the total participants (50%, n=20) indicated water shortage to last for 2 or more days predominantly seen during the months of April-June. More than half (57%, n=25) of the study participants agreed that there were no problems in supplied water. The most common problems faced during the water supply included unclean water (28%, n=11), irregular water supply (12%, n=5) and bad odor (7%, n=3). Only 33% (n=13) of the study participants cleaned water containers daily. Less than half of the participants (45%, n=18) had toilet facilities inside their households, while an equal number used community toilets. Most of the participants (90%, n=36) had access to flush/pour flush to piped sewer system type of toilet facility (Table 3).

**Table 3:** Water and sanitation facility & uses

Facility and Uses	
Variables	Results
**Water Facility**	
**Main source for water procurement**	
Piped water in yard/plot	45%(n=18)
Tube well/bore well	30% (n=12)
Piped water in yard/plot and tube well/bore well	18% (n=7)
Piped water in dwelling	5% (n=2)	
Piped water in dwelling and piped water in yard/plot	2% (n=1)	
**Supplier of water**	
Public	53% (n=21)	
Private	27% (n=11)	
Both	18% (n=7)	
**Time consumed in fetching water from source, ≤30 minutes**	95% (n=38)	
**Distance of water source**		
With in household	48% (n=19)	
≤20 metes	40% (n=16)	
>20 meters	12% (n=5)	
**Family member fetching water from the source**		
Female>15 years	93% (n=37)	
Male <15 years	15% (n=6)	
Male>15 years	15% (n=6)	
Female <15 years	10% (n=4)	
**Daily water needs fulfillment**		
Yes	88% (n=35)	
No	12% (n=5)	
**Frequency of water shortage faced in a year**		
Twice or more	40% (n=16)	
No shortage	35% (n=14)	
Once	15% (n=6)	
Sometimes	5% (n=2)	
**Average time period of shortage**		
Two or more than two days	53% (n=21)	
One day	13% (n=5)	
**Maximum scarcity faced in the period of the year**		
No shortage through the year	43% (n=17)	
April-June	35% (n=14)	
No specific period	12% (n=5)	
Shortage throughout the year	10% (n=4)	
**Timing of water supply**		
Morning and Evening	75% (n=30)	
Round the clock	23% (n=9)	
**Water storage container**		
Narrow mouth closed container	63% (n=25)	
Wide mouth closed container	50% (n=20)	
Wide mouth open container	4% (n=2)	
**Frequency of cleaning water container**		
Daily	33% (n=13)	
More than a day	33% (n=13)	
Before fetching water	25% (n=10)	
When dirty	7% (n=3)	
Sometimes	2% (n=1)	
**Problems faced in supplied water**		
None	63% (n=25)	
Unclean	28% (n=11)	
Irregular supply	12% (n=5)	
Bad odour	7% (n=3)	
**Sanitation facility**		
**Toilet facility**		
Household	45% (n=18)	
Community	45% (n=18)	
Shared	10% (n=4)	
**Toilets types**		
Flush/pour flush; to piped sewer system**	90% (n=36)	
Elsewhere ***	10% (n=4)	
**Type of drainage**		
Open	83% (n=33)	
Closed	17% (n=7)	

* Some of the variables are in multiple responses

** Piped sewer system: system of sewer pipes, also called sewerage is designed to collect human excreta and waste water and remove them from household environment (WHO/UNICEF, 2006).

*** Elsewhere: Flush poured flush where excreta been deposited in or nearby household environment (not into a pit septic tank or sewer). Excreta may be flushed street yard/plot, open sewer, a ditch, a drainage way or other location (WHO/UNICEF, 2006).

**Figure 1:**
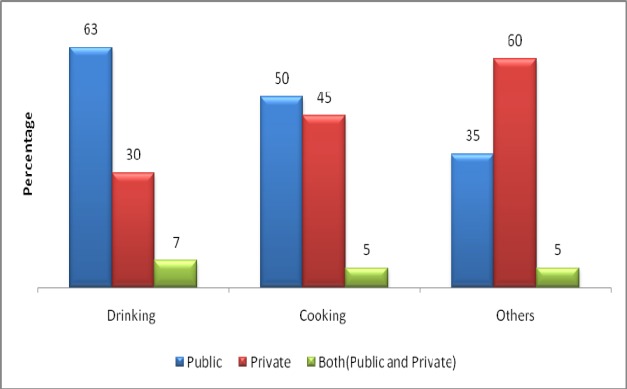
Comparison of daily water consumption from different water sources for drinking, cooking and other (washing, ablution, cleaning and miscellaneous) usage

Results showed that more than half of the participants (63%) used public supply water for drinking, 30% used private supply water and 7% used both. Half of the participants used public supply water for cooking. Three out of five participants used private supply of water for other (ablutions, washing & cleaning) purposes (Figure 1). The average amount of water consumed in a day for drinking, cooking and other purposes (ablutions, washing clothes, house cleaning and miscellaneous) was found to be about 16, 18 and 318 liters, respectively.

No significant association was found between independent variables including age, gender, education and occupation and the outcome daily water need fulfillment (Table 4).

**Table 4:** Association of socio-demographic variables with daily water needs fulfillment

	Daily water need fulfillment		p-value
	Yes	No	
**Age group (years)**			
≤40	86% (n=25)	14% (n=4)	0.688
>40	91% (n=10)	9% (n=1)	
**Gender**			
Male	91% (n=10)	9% (n=1)	0.688
Female	86% (n=25)	14% (n=4)	
**Education**			
Educated	85% (n=17)	15% (n=3)	0.633
Uneducated	90% (n=18)	10% (n=2)	
**Occupation**			
Unemployed	85% (n=23)	15% (n=4)	0.604
Skilled worker	100% (n=6)		
Others	86% (n=6)	14% (n=1)	

Age was significantly associated with methods that were being used to make water safe (p=0.041). No other variables were significantly associated with methods used to make water safe (Table 5).

**Table 5:** Association of socio-demographic variables with water treatment practices

	Current methods being used to make water safe			p-value
	Nothing	Filter	Boiling	
**Age group (years)**				
≤40	79% (n=23)	7% (n=2)	14% (n=4)	0.041
>40	64% (n=7)	36% (n=4)		
**Gender**				
Male	55% (n=6)	27% (n=3)	18% (n=2)	0.184
Female	83% (n=24)	10% (n=3)	7% (n=2)	
**Education**				
Educated	75% (n=15)	15% (n=3)	10% (n=2)	1
Uneducated	75% (n=15)	15% (n=3)	10% (n=2)	
**Occupation**				
Unemployed	81% (n=22)	15% (n=4)	4% (n=1)
Skilled worker	33% (n=2)	33% (n=2)	33% (n=2)	0.075
Others	86% (n=6)		14% (n=1)

Water quality: 53% (n=21) of the water samples collected from the households of participants showed evidence of bacteriological contamination. Majority of water contamination was found to be in the household’s samples with water stored from tube well as the drinking water source.

## 4. Discussion

Provision of accessible, affordable and acceptable safe drinking water facility to each and every individual of the world; regardless of cast, ethnicity, gender, socio-economic status and geographical location is essential. Although there is an increase in coverage of drinking water facility decade by decade, sharp increase in urban population in an unorganized manner has started creating an imbalance between demand and supply of water, and the gap is expected to increase in future (UN MDGs, 2013). Individuals living in slums are likely to be affected most from this imbalance.

This pilot study attempted to assess the present situation of water and sanitation facilities, attitude and practices of the individuals living in urban slums of New Delhi. Additionally it attempted to assess the water quality of stored water among the same households.

The ‘Core questions on drinking-water and sanitation for household surveys’ by WHO-UNICEF, used in the study, was developed to make survey data on drinking-water and sanitation needs of the population comparable and accurate across nations. Extensive use of this set of harmonized questions has been advocated for evidence-based decisions and rightly directed efforts (WHO/UNICEF, 2006).

Assessment of bacteriological contamination was done by using H_2_S vials. The use of H_2_S vials is imminent not only to serve the purpose of initial screening of contamination but is also an effective tool for generating awareness amongst the community to consume only safe drinking water and maintain (Government of India Ministry of Drinking Water and Sanitation [GoI MDWS], 2013). These vials are simple, reliable, and a low cost and easy-to-use alternative to laboratory testing in remote and resource-poor settings particularly valuable in instances of intermittent contamination (Wright et al., 2012).

45% of the individuals were consuming water from tube well/bore well run by private suppliers or community representatives. In the current study, April-June was found to be the most critical time of water shortage in the community and in almost half of the cases it was seen that average time period of water shortage was found to be two or more than two days. A study on water quality of groundwater resources showed that the water quality index of bore well, dug well and hand pump declined in post monsoon season (Rajankar et al., 2009).

Half of the respondents had to move out of their houses to fetch water with females above 15 years being majorly responsible for doing the same. Majority of the burden is on women, who are largely responsible for fetching water from distant sources (Venkatachalam, 2011). Despite the water supply timings being in the morning and evening the role of adult male partners was found to be limited in fetching the water. Similar findings were found in a study on the role of informal water markets in urban water supply, which revealed that 81% of families fetching water from a distant source and women were more responsible for fetching water in comparison to males (Poulos et al., 2012). Girl child is four times more responsible for filling water from distant source than male child (Poulos et al., 2012).

Most of the respondents felt that water was safe for drinking while 95% (n=38) of the participants felt that level of water quality can affect health and more than two-third of the participants (83%; n=33) felt that unclean water can cause gastro intestinal tract infection or disorder. Study on consumer preferences for household water treatment products showed that 15% of the households used boiling, 26% of them used filtration and less than 1% used chemical treatment for drinking water (Wright et al., 2012). In the current study, three-fourth of the respondents were not using any method to treat the water and 73% (n=22) felt that water is already clean so there is no need to treat it. In contrast, the result of water sample collected from households showed that 53% of the samples were contaminated. Suthar S, 2011 showed that the potable water samples from 78% of the town/villages showed *E.coli* contamination (Suthar, 2011).

On assessing the situation and practices related to sanitation it was found that only 45% (n=18) of the individuals had access to toilets inside the households, so more than half of the pilot study population had to go out of the houses for defecation. In past study, it was found that there were 2.5 billion people who lacked access to an improved sanitation facility (WHO/UNICEF, 2013). Of these, 761 million use public or shared sanitation facilities and another 693 million use facilities that do not meet minimum standards of hygiene (unimproved sanitation facilities). The remaining one billion (15% of the world population) still practice open defecation [WHO/UNICEF, 2013]. Majority of the participants washed their hands both before handling of the food and eating, 88% (n=35) of the participants washed their hands after defecation and 75% (n=30) of them wash their hands after eating. 78% (n=31) of the participants agreed that washing hands improves hygiene and 75% (n=30) of them agreed that it prevents infection.

There were several limitations of the current study and included smaller sample size, cross sectional design and limited geographical location so the results of the study cannot be generalized. Since the positive predictive value of H_2_S test also depends on the frequency of water contamination, a longitudinal study may be warranted to test the water quality of stored water including the sources to avoid needless concern and counteraction (Wright et al., 2012).

Impact on the performance of children in school, who are involved in helping the family for fetching water needs to be explored. Future research is warranted to design and develop family centered interventions that are aimed to facilitate improvement of water, sanitation and hygiene related knowledge, attitude and practice among various stake holders to improve outcomes in diverse settings.
